# Early-Stage B-cells Predict Relapse After Rituximab Treatment in Patients With Membranous Nephropathy

**DOI:** 10.1016/j.ekir.2026.106365

**Published:** 2026-02-19

**Authors:** Yousra Cheddadi, Mounir El Maï, Vesna Brglez, Sarah Nahon Carzo, Marion Cremoni, Maxime Teisseyre, Barbara Seitz-Polski

**Affiliations:** 1 Laboratoire d’Immunologie et de Thérapie Cellulaire, Centre Hospitalier Universitaire de Nice, Nice, France; 2 Centre de Référence Maladies Rares Syndrome Néphrotique Idiopathique et Glomérulonéphrite Extra-Membraneuse, Centre Hospitalier Universitaire de Nice, Nice, France; 3 Université Côte d’Azur, CNRS, INSERM, IRCAN, Nice, France

## Introduction

Membranous nephropathy (MN) is an autoimmune kidney disease and the leading cause of nephrotic syndrome in adults. B-cells play a pivotal role in the pathogenesis of MN through the generation of autoantibodies. Approximately 70% of patients with MN have autoantibodies targeting the phospholipase A2 receptor 1 (PLA2R1).^1^ The discovery of these autoantibodies has transformed the management of MN, promoting the use of targeted B-cell-depleting therapies.^2^ Rituximab selectively depletes B-cells and has become a cornerstone of treatment. Several studies have demonstrated its effectiveness in achieving clinical remission in MN.^3^^,^^4^ Although many patients achieve long-lasting remission, some relapse within months. This emphasizes the importance of improving our understanding of the mechanisms underlying variable treatment responses. As B-cells are the main source of autoantibodies, monitoring their reconstitution after rituximab-induced depletion is a promising way of understanding the immunological factors of treatment efficacy and relapse. Some studies have demonstrated a correlation between B-cell reconstitution and relapse following treatment with rituximab in disorders such as idiopathic nephrotic syndrome, myasthenia gravis, and rheumatoid arthritis but not in MN.^5^, ^6^, ^7^ This study aimed to determine whether B-cell reconstitution can predict relapse in MN, and to characterize the specific B-cell subpopulations involved.

The full protocol for this study is provided in the Supplementary Methods.

## Results

Thirty-nine patients were included from a randomized, multicenter, prospective trial personalized medicine for membranous nephropathy (PMMN) (NCT03804359) comparing the efficacy of 2 rituximab-based treatment strategies for phospholipase A2 receptor 1-associated MN (Supplementary Figure S1).^8^^,^^9^ Of these patients, 36 received rituximab, and 3 achieved spontaneous remission. The depletion and reconstitution of B-cell subpopulations following rituximab treatment were assessed using flow cytometry (see the Supplementary Data for methods). Patient characteristics are summarized in Table 1 and Supplementary Table S1. Following rituximab treatment, 19 patients achieved and maintained clinical remission, including 8 who achieved complete remission and 11 who achieved partial remission. Nine patients failed to achieve remission, and 8 experienced a relapse comprising 2 clinical and 6 immunological relapses. Immunological relapse was defined as an increase in anti-phospholipase A2 receptor 1 antibody titers >14 RU/mL after achieving immunological remission (< 14 RU/mL). Among patients who experienced immunological relapse, 5 achieved partial clinical remission and 1 achieved complete clinical remission. Of the 2 patients who subsequently experienced clinical relapse, 1 was retreated after month-6 with rituximab, whereas the other received obinutuzumab because of immunization against rituximab.

**Table 1:** Characteristics of relapsing and nonrelapsing patients during the follow-up period

Parameter	All (*n* = 27)	Nonrelapsers (*n* = 19)	Relapsers (*n* = 8)	*P*-value
Creatinine (μmol/L), mean ± SD				
M3	112.1 ± 35.7	113.6 ± 36.6	108.5 ± 35.5	0.5
M6	112.3 ± 35.4	113.2 ± 31.4	110.6 ± 45.2	0.5
M12	107.9 ± 29.7	107.5 ± 26.5	108.6 ± 37.8	0.8
M18	101.8 ± 24.4	100.3 ± 26.22	104.0 ± 22.9	0.6
Albumin (g/L), mean ± SD				
M3	30.4 ± 6.2	32.3 ± 5.6	26.5 ± 6.2	0.04
M6	33.1 ± 6.8	34.6 ± 5.6	29.7 ± 8.3	0.1
M12	37.6 ± 4.9	38.8 ± 4.4	35.1 ± 5.4	0.04
M18	38.7 ± 3.6	40.6 ± 2.9	35.9 ± 2.5	0.002
Proteinuria (g/g), mean ± SD				
M3	4.44 ± 3.17	4.40 ± 3.66	4.51 ± 1.80	0.6
M6	3.68 ± 3.39	3.26 ± 3.27	4.50 ± 3.70	0.3
M12	1.92 ± 2.44	1.80 ± 2.88	2.19 ± 1.17	0.2
M18	1.42 ± 1.42	0.91 ± 1.02	2.20 ± 1.64	0.04
Anti-PLA2R1 (RU/ml), median (IQR)				
M3	0.00 (0.00–6.00)	0.00 (0.00–3.50)	6.00 (3.00–7.00)	0.03
M6	0.00 (0.00–14.50)	0.00 (0.00–4.00)	14.50 (3.25–37.25)	0.007
M12	0.00 (0.00–2.25)	0.00 (0.00–0.00)	9.50 (0.00–20.50)	0.005
M18	0.00 (0.00–15.75)	0.00 (0.00–0.00)	16.00 (15.00–23.00)	< 0.001
Residual serum RTX level (μg/ml), median (IQR)				
M3	0.00 (0.00–0.00)	0.00 (0.00–0.00)	0.00 (0.00–0.00)	0.5
Anti-RTX, *n* (%)				
M3^a^				> 0.99
Yes, *n* (%)	0 (0)	0 (0)	0 (0)	
No, *n* (%)	25 (100)	18 (100)	7 (100)	
M6^a^				0.28
Yes, *n* (%)	5 (20)	2 (11.8)	3 (37.5)	
No, *n* (%)	20 (80)	15 (88.2)	5 (62.5)	
M12^b^				> 0.99
Yes, *n* (%)	6 (23.1)	4 (22.2)	2 (25.0)	
No, *n* (%)	20 (73.9)	14 (77.8)	6 (75.0)	
M18^c^				> 0.99
Yes, *n* (%)	2 (10)	1 (7.7)	1 (14.3)	
No, *n* (%)	18 (90)	12 (92.3)	6 (85.7)	
Time to immunological remission after RTX (mos), mean ± SD	4.7 ± 4.2	5.0 ± 4.9	4.2 ± 3.0	> 0.99
Time to clinical remission after RTX (mos), mean ± SD	8.6 ± 5.5	8.5 ± 5.8	8.9 ± 5.4	0.8
Clinical remission after RTX, *n* (%)				0.2
CCR	9 (33.3)	8 (42.1)	1 (12.5)	
PCR	18 (66.7)	11 (57.9)	7 (87.5)	

CCR, complete clinical remission; IQR, interquartile range; mos, months; PCR, partial clinical remission; PLA2R1, phospholipase A2 receptor 1; RTX, rituximab; SD, standard deviation.

a *n* = 25.

b *n* = 26.

c *n* = 20.

**Figure 1:**
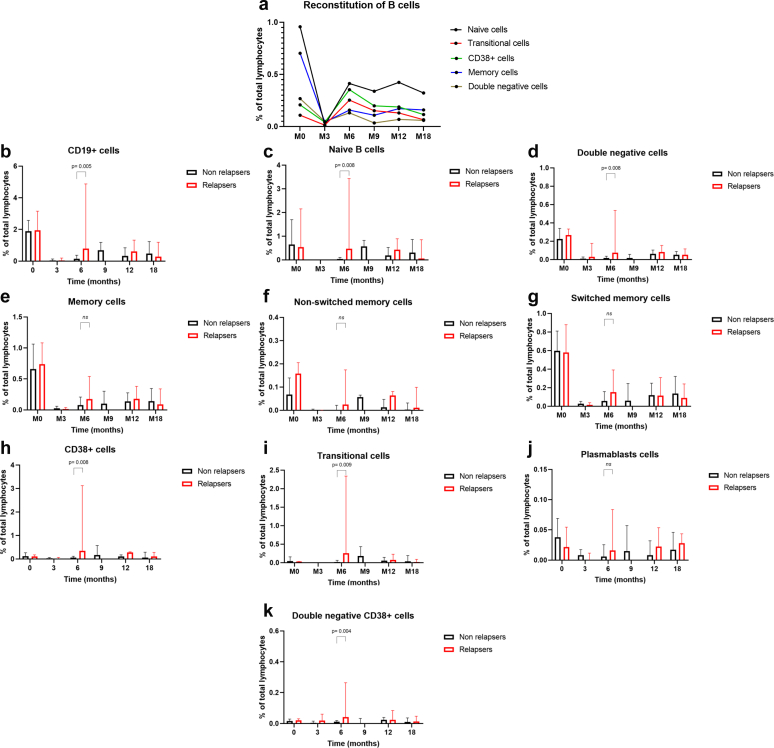
Reconstitution of B-cells over time. To characterize B-cell subsets, peripheral blood mononuclear cells were stained with fluorochrome-conjugated monoclonal antibodies (BD Biosciences) directed against the following antigens: CD45 (V500-C), CD3 (APC), CD19 (APC-H7), CD27 (BV421), IgD (PE), CD38 (BV711), CD4 (BV605), and CD8 (PE), as well as 7-AAD (Miltenyi Biotech). The stained cells were then analyzed using multicolor flow cytometry (BD FACS Lyric). The subsets of gated CD19^+^ cells were identified based on surface marker expression as follows: naïve (CD19+CD27-IgD+), nonswitched memory (CD19+CD27+IgD+), switched memory (CD19+CD27+IgD-), and double negative (CD19+CD27-IgD-); and within the CD19+CD38++ population, we distinguished transitional cells (CD19+CD38++CD27-IgD+), plasmablasts (CD19+CD38++CD27+IgD-), and double-negative CD38^+^ cells (CD19+CD38++CD27-IgD-). B-cell subsets were expressed as a percentage of the total lymphocyte count. (a) B-cell subpopulations were assessed in 39 patients at baseline and in 36 patients at 3 (M3), 6 (M6), 9 (M9), 12 (M12), and 18 (M18) months following the first rituximab infusion. The 3 patients who did not receive treatment were excluded from the follow-up. Complete depletion of B-cells was observed in all patients at M3 post-rituximab for all B-cell subpopulations. CD19^+^ cells reappeared 6 months after rituximab infusion. Naive cells re-emerged the most among B-cells, followed by CD38^+^ cells, transitional cells and finally memory cells. Data are shown as mean values (dots). (b–k) B-cell subpopulations at baseline and at subsequent time points were compared between relapsing patients (*n* = 8) and nonrelapsing patients (*n* = 19); the 2 patients who received additional anti-CD20 infusions were excluded from subsequent analyses. Data are shown as medians and interquartile range (IQR). *P*-values were calculated by comparing the median values of each cell subpopulation between relapsing and nonrelapsing patients using a nonparametric, unpaired Mann–Whitney U test.

Complete depletion of all B-cell subpopulations was observed in all patients 3 months (M3) after rituximab treatment (Figure 1). CD19^+^ cells began to reappear at 6 months (M6), most notably naïve B-cells, followed by CD38^+^, transitional, and memory cells. At baseline, no significant differences were observed between patients and healthy donors in the median proportions of naïve, total memory, nonswitched memory, CD38^+^, or transitional B-cells. However, patients showed a trend toward higher levels of total B-cell (median 1.79% vs. 0.65%, *P* = 0.05) and double-negative B-cells (0.22% vs. 0.12%, *P* = 0.06) (Supplementary Figure S2). During follow-up, baseline median B-cell levels were not regained in any subpopulation, except for CD38^+^ and transitional B-cells at months 6, 9, 12, and 18 (Supplementary Figure S2). The proportion of naïve cells was significantly lower than at baseline after rituximab, which suggests that the maturation of transitional cells into naïve cells was probably still ongoing (Figure 1 and Supplementary Figure S2). The memory compartment remained significantly reduced throughout the follow-up period compared to baseline (Supplementary Figure S2). This pattern has previously been observed after rituximab treatment in MN and aligns with the physiological development of B-cells, whereby memory cells are the last to re-emerge.^4^ These results are also consistent with those in the study by Rosenzwajg *et al.*,^10^ which reported that CD19^+^ cell counts had not returned to baseline by 6 months, mostly because of a lack of reconstitution of nonswitched memory B-cells. Using Spearman’s correlation analysis, we found a significant correlation between the count of plasmablasts and the anti-phospholipase A2 receptor 1 antibody titer at M12 (*P* = 0.015, data not shown). At M6, the proportions of total CD19^+^, naïve, double-negative CD19^+^, and CD38^+^ cells were significantly higher in relapsing patients than in nonrelapsing patients (CD19^+^: 0.80% vs. 0.15%, *P* = 0.005; naïve: 0.47% vs. 0.04%, *P* = 0.008; double-negative CD19^+^: 0.076% vs. 0.018%, *P* = 0.008; CD38^+^: 0.35% vs. 0.06%, *P* = 0.008). Among CD38^+^ cells, both transitional CD38^+^ and double-negative CD38^+^ subsets were significantly higher in relapsing patients than in nonrelapsing patients (0.25% vs. 0.02%, *P* = 0.009; and 0.04% vs. 0.01%, *P* = 0.004; respectively) (Figure 1). The optimal cut-offs for predicting relapse, expressed as a percentage of total lymphocytes, were found to be as follows: 0.44% for CD19^+^ cells, 0.18% for naïve cells, 0.028% for double-negative CD19^+^ cells, 0.12% for CD38^+^ cells, 0.072% for transitional cells CD38^+^, and 0.024% for double-negative CD38^+^ cells (Supplementary Figure S3). Patients exceeding these thresholds were at a significantly higher risk of relapse within 18 months (all *P* < 0.005) (Supplementary Figure S3). When considering only patients with immunological relapse, the proportions of total CD19^+^, double-negative CD19^+^, and double-negative CD38^+^ cells were higher in relapsing patients than in nonrelapsing patients (CD19^+^: 0.54% vs. 0.15%, *P* = 0.04; double-negative CD19^+^: 0.043% vs. 0.018%, *P* = 0.047; and double-negative CD38^+^: 0.03% vs. 0.01%, *P* = 0.046). Naïve, CD38^+^, and transitional CD38^+^ cells also tended to be higher in patients with immunological relapse (naïve: 0.29% vs. 0.04%, *P* = 0.055; CD38^+^: 0.16% vs. 0.06%, *P* = 0.077; transitional CD38^+^: 0.12% vs. 0.02%, *P* = 0.07). In contrast to idiopathic nephrotic syndrome and myasthenia gravis, no significant differences were observed between relapsers and nonrelapsers in the reconstitution of total memory B-cells or nonswitched or switched memory B-cells at any time point.^5^^,^^6^ However, memory cell recovery occurred earlier in relapsers, whose median percentages did not differ from baseline at M6 (*P* = 0.4), M12 (*P* = 0.2) or M18 (*P* = 0.1). In contrast, nonrelapsers showed persistently lower proportions of memory B-cells than at baseline at M6 (*P* = 0.0005), M12 (*P* = 0.007), and M18 (*P* = 0.007). Furthermore, from 12 months post-rituximab onwards, the proportions of memory and double-negative cells remained stable in patients who relapsed, whereas they declined in those who did not (Supplementary Figure S4). Among patients who relapsed, the percentage of switched memory cells was higher during relapse than during remission (Supplementary Figure S4). This study is, to the best of our knowledge, the first to demonstrate the prognostic value of B-cell subpopulations in predicting relapse in patients with MN. Our findings suggest that the proportion of total B-cells, naïve cells, double-negative cells, and CD38^+^ cells at M6 may be useful in predicting relapse. These results are consistent with those of a previous study in rituximab-treated rheumatoid arthritis patients, which showed that repopulation of total B-cells and transitional cells was associated with relapse.^7^ In the study by Rosenzwajg *et al.*,^10^ no association was observed between B-cell subsets and treatment response in MN. However, patients who clinically responded to rituximab exhibited a significantly lower percentage of regulatory T cells at baseline compared to nonresponders and a significantly increased percentage at day 8. Our results imply that the early reconstitution of immature B-cells could trigger a relapse, indicating that the autoimmune response is still active. It can also be hypothesized that these immature and naïve B-cells subsequently differentiate into long-lived autoreactive plasma cells, thereby sustaining disease activity. Double-negative B-cells, which play diverse roles in infections, cancers, and autoimmune diseases, also emerged as potential predictors of relapse. Overall, monitoring B-cells could help to identify patients at high risk of relapse who might benefit from early retreatment with rituximab. Our findings also demonstrate the prognostic value of CD38^+^ cells and support the development of anti-CD38 monoclonal antibodies for treating MN.^11^

## Disclosure

The authors declare no conflicts of interest.

## Supplementary Material

Supplementary File (PDF) **Supplementary Methods. Supplementary References. Figure S1.** Flowchart of the study. Figure S2. Multicolor flow cytometry analysis of B cell subpopulations at baseline and different time. Figure S3. Determination of the optimal cut-offs for predicting relapse. Figure S4. Reconstitution of memory and double-negative cells among relapsing and non-relapsing patients. Table S1. Baseline characteristics of the patients in the study.
