# Supplementary material for: Early-Stage B-cells Predict Relapse After Rituximab Treatment in Patients With Membranous Nephropathy
Source: Kidney Int Rep. 2026 Feb 19;11(5):106365. doi: 10.1016/j.ekir.2026.106365 (PMC13022648; doi:10.1016/j.ekir.2026.106365)
Supplement: Supplementary File (PDF) — Supplementary Methods. Supplementary References. Figure S1. Flowchart of the study. Figure S2. Multicolor flow cytometry analysis of B cell subpopulations at baseline and different time. Figure S3. Determination of the optimal cut-offs for predicting relapse. Figure S4. Reconstitution of memory and double-negative cells among relapsing and non-relapsing patients. Table S1. Baseline characteristics of the patients in the study. [file mmc1.pdf]

Supplementary data

Methods

Study design

This is an ancillary study from a randomized, open-label, multicentre, prospective trial: Personalised Medicine for Membranous Nephropathy (PMMN), registered under NCT03804359. This study compared the efficacy of two therapeutic strategies (standard vs. personalised) in achieving clinical remission 12 months after inclusion in patients with PLA2R1-associated MN. A retrospective analysis was conducted on prospectively collected samples from all patients in the standard arm (n = 29) and from those in the personalised arm who were treated with rituximab six months after inclusion (n = 10). Patients were enrolled at several French hospitals between November 2019 and October 2022, and were observed for two years following enrolment.

Inclusion criteria were: aged 18 years or more; anti-PLA2R1 activity detected by enzyme-linked immunosorbent assay (ELISA) or immunofluorescence assay; nephrotic syndrome defined by proteinuria > 3.5 g/24 h (or urinary protein/creatinine ratio (UPCR) > 3.5 g/g) and serum albumin < 30 g/L; estimated glomerular filtration rate (eGFR with CKD-EPI formula) >30 mL/min/1.73 m^2; symptomatic treatment according to kidney disease improving global outcome (KDIGO) guidelines: maximal tolerated dose of non-immunosuppressive antiproteinuric treatment (NIAT, angiotensin-converting enzyme inhibitor and/or angiotensin 2 receptor blockers); medical insurance; and signed informed consent.

Exclusion criteria were: secondary MN (MN related to cancer, infections, systemic lupus erythematous, drugs); anti-PLA2R1 antibodies not confirmed by central analysis; pregnancy or breastfeeding; immunosuppressive treatment in the last six months; cancer under treatment; complicated nephrotic syndrome that would require early immunosuppressive treatment (thrombosis, acute renal failure); severe infections or active hepatitis B; hypersensitivity to the active substance or to any of the other excipients; severely immunocompromised state; severe heart failure (New York Heart Association Class IV) or severe uncontrolled cardiac disease; presence of anti-rituximab antibodies; unable to give an informed consent.

We divided patients into four groups: non-relapsers (n=19), relapsers (n=8), those who did not achieved clinical remission (n=9) and those who did not receive rituximab treatment (n=3) (Figure S1).

Remission was defined as either partial, characterised by proteinuria of less than 3.5 g/g with a reduction of at least 50% from baseline, serum albumin of at least 30 g/L and an increase in serum

creatinine of no more than 20% from baseline, or complete, characterised by proteinuria of less than 0.3 g/g and serum albumin of at least 35 g/L.

Patients who received a new immunosuppressive treatment during follow-up were considered not to have achieved clinical remission. Relapse was a composite criterion of clinical or immunological relapse. Clinical relapse was defined as the recurrence of proteinuria >3.5 g/g after remission. Immunological relapse was defined as an increase in anti-PLA2R1 antibodies >14 RU/mL after achieving immunological remission (anti-PLA2R1 <14 RU/mL).

Sample

The sample collected immediately before the first rituximab infusion was considered as baseline. Patients were then scheduled at three, six, nine, 12- and 18-months post-treatment.

Written informed consent was obtained from all participants. Samples were collected according to the to the PMMN protocol approved by the local ethics committee, and the study adhered to the declaration of Helsinki. Blood samples were shipped on the same day to the Nice Immunology Laboratory for analysis, including the quantification of anti-PLA2R1 antibodies and isolation of peripheral blood mononuclear cells (PBMCs).

Flow cytometry

To characterise B-cell subsets, PBMCs were stained with fluorochrome-conjugated monoclonal antibodies (BD Biosciences) directed against the following antigens: CD45 (V500-C), CD3 (APC), CD19 (APC-H7), CD27 (BV421), IgD (PE), CD38 (BV711), CD4 (BV605) and CD8 (PE), as well as 7-AAD (Miltenyi Biotech). The stained cells were then analyzed using multicolour flow cytometry (BD FACS Lyric). The subsets of gated $CD19^{+}$ cells were identified based on surface marker expression as follows: naïve (CD19+CD27-IgD+), non-switched memory (CD19+CD27+IgD+), switched memory (CD19+CD27+IgD-), double negative (CD19+CD27-IgD-) and within the CD19+CD38++ population, we distinguished transitional cells (CD19+CD38++CD27-IgD+), plasmablasts (CD19+CD38++CD27+IgD-), and double-negative CD38+ cells (CD19+CD38++CD27-IgD-). B-cell subsets were expressed as a percentage of the total lymphocyte count.

All analyses were performed using FACSSuite v1.5 software. Gated events (100,000) on living lymphocytes (7-AAD negative) were analyzed for each sample.

Laboratory measurements

Serum levels of total IgG anti-PLA2R1 antibodies were measured using an ELISA test developed by EUROIMMUN AG (Lübeck, Germany). Participants were considered anti-PLA2R1-positive if their levels were >14 RU/mL.

Residual serum rituximab levels were measured by ELISA according to the manufacturer's instructions (LISA-TRACKER Duo Rituximab, Theradiag, Croissy-Beaubourg, France). The limit of detection for residual rituximab levels was >2 µg/mL. Anti-rituximab antibodies were detected using an ELISA test according to the manufacturer's instructions (LISA-TRACKER Duo Rituximab, Theradiag, Croissy-Beaubourg, France). Anti-rituximab antibodies were considered present if levels were >10 ng/mL.

Statistical analyses

Depending on whether the variable had a normal distribution, quantitative variables were expressed as mean ± standard deviation (SD) or median and interquartile ranges. Normality was assessed using the Shapiro–Wilk test. The comparison of quantitative variables was performed using an unpaired t-test if the variable was normally distributed, or a non-parametric Mann–Whitney test if it was not. Qualitative variables were expressed as numbers and percentages, and compared using the chi-squared or Fisher's exact test, as appropriate. The predictive value of each B cell subset for relapse was analyzed using ROC analysis, the Kaplan–Meier method, and the log-rank test. We analyzed the correlation between B-cell counts and anti-PLA2R antibody titers using Spearman's correlation analysis. All p-values were two-sided and considered statistically significant at $p < 0.05$. Analyses were performed using GraphPad Prism 8.4.

Table S1: Baseline characteristics of the patients in the study.

Parameters	All (n=39)	Non-relapsers (n=19)	Relapsers (n= 8)	No remission (n=9)	Non-treated (n=3)
**Demographics**					
Age, mean ± SD	62.9 ± 12.3	63.2 ± 11.4	63.6 ± 11.8	58.4 ± 15.2	72.3 ± 11.1
Sex, n (%)					
Male	28 (71.8)	11 (57.9)	7 (87.5)	8 (88.8)	2 (66.7)
Female	11 (28.2)	8 (42.1)	1 (12.5)	1 (11.1)	1 (33.3)
**Clinical characteristics**					
Creatinine (µmol/L), median (IQR)	113.0 (91.0-142.0)	119.6 (85.0-145.0)	110.5 (85.5-140.8)	129.0 (101.5-143.5)	91.0 (86.0-105.2)
Albumin (g/L), mean ± SD	24.5 ± 5.3	24.9 ± 6.2	21.7 ± 5.5	22.2 ± 4.8	28.9 ± 6.2
Proteinuria (g/g), mean ± SD	6.9 ± 2.8	6.6 ± 2.6	7.2 ± 3.3	6.9 ± 2.9	9.0 ± 2.6
Anti-PLA2R1 (RU/mL), median (IQR)	73.0 (19.5-158.5)	73.0 (11.0-148.0)	99.0 (29.75-188.5)	138.0 (26.0-461.5)	25.0 (19.0-29.0)
Anti-RTX, n (%)					
0	38 (97.4)	19 (100)	7 (87.5)	9 (100)	3 (100)
NA	1 (2.6)	0 (0)	1 (12.5)	0 (0)	0 (0)
MN history					
First course, n (%)	24 (61.5)	9 (47.3)	6 (75.0)	6 (66.7)	3 (100)
Relapse, n (%)	15 (38.5)	10 (52.6)	2 (25.0)	3 (33.3)	0 (0)
Spreading, n (%)					
Yes	12 (30.8)	4 (21.1)	2 (25.0)	4 (44.4)	2 (66.7)
No	27 (69.2)	15 (78.9)	6 (75.0)	5 (55.6)	1 (33.3)
**B cell subsets, (% of total lymphocytes), median (IQR)**					
$CD19^+$	1.79 (0.77-2.62)	1.88 (0.87- 2.57)	1.95 (0.52-3.15)	1.05 (0.34 2.58)	2.16 (1.25-2.88)
Naive	0.64 (0.13-1.64)	0.65 (0.12-1.69)	0.54 (0.13-2.15)	0.30 (0.07-1.47)	0.74 (0.65-1.26)
Double negative	0.22 (0.11-0.33)	0.22 (0.09-0.34)	0.27 (0.15-0.33)	0.13 (0.07-0.34)	0.29 (0.21-0.53)

Memory cells	0.63 (0.22-1.06)	0.66 (0.17-1.06)	0.74 (0.12-1.08)	0.39 (0.20-1.72)	0.61 (0.39-1.08)
Non-switched memory	0.09 (0.03-0.17)	0.07 (0.04-0.14)	0.16 (0.03-0.20)	0.10 (0.03-0.27)	0.21 (0.12-0.21)
Switched memory	0.54 (0.15-0.84)	0.60 (0.13-0.81)	0.58 (0.09-0.88)	0.28(0.17-0.83)	0.40 (0.11-1.49)
$CD38^+$	0.12 (0.05 -0.24)	0.12 (0.05-0.26)	0.11 (0.06-0.17)	0.13 (0.02-0.29)	0.19 (0.11-0.46)

PLA2R1, phospholipase A2 receptor; RTX, rituximab; MN, membranous nephropathy; NA, not available.

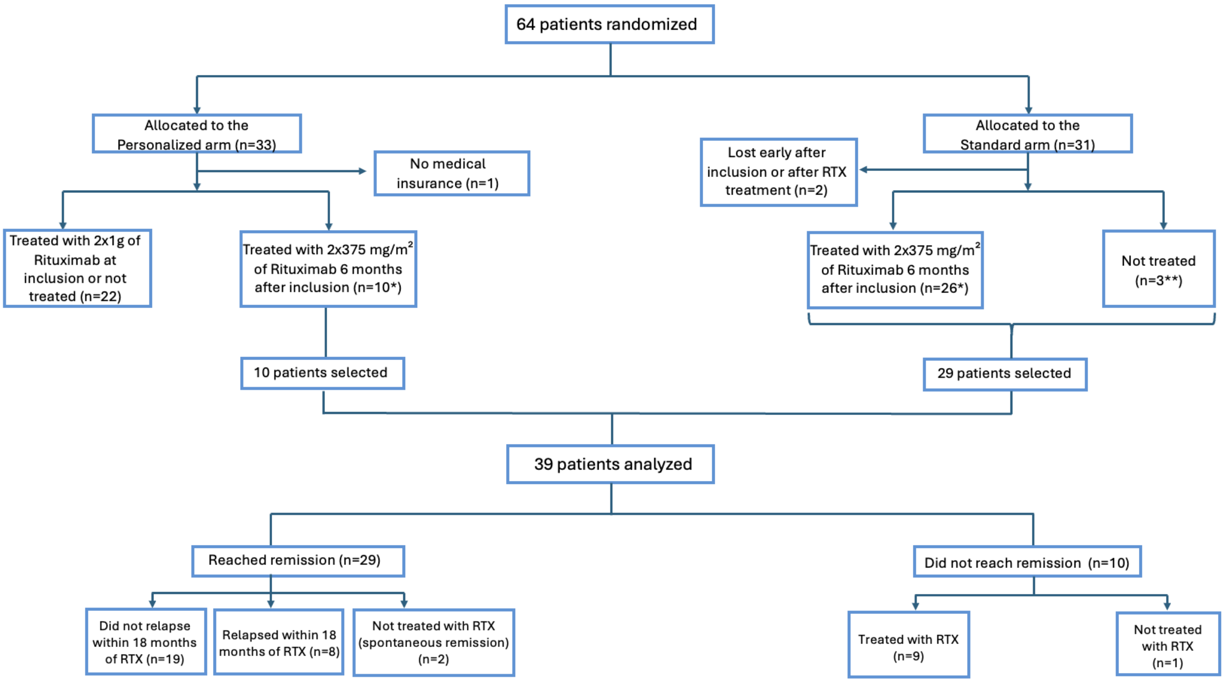

Figure S1: Flowchart of the study.

Of the 64 patients randomized in the PMMN study, 39 from the standard (n = 29) and personalised (n = 10) arms were analyzed here. In the standard arm, 26 patients received two infusions of rituximab (2x375 mg/m² at a one-week interval after six months of non-immunosuppressive antiproteinuric treatment), while three patients were not treated. In the personalised arm, 10 patients received two infusions of rituximab (2x375 mg/m² at a one-week interval) after six months of non-immunosuppressive antiproteinuric treatment. Patients from the personalised arm who were treated immediately at inclusion or who did not receive rituximab treatment were not included in this study (n = 22).

We divided the patients into four groups: non-relapsers (n = 19), relapsers (n = 8), patients who did not reach remission (n = 9), and patients who did not receive rituximab treatment (n = 3).

*Three patients from the standard group and one patient from the personalised group received two 1 g rituximab infusions due to a worsening of their clinical symptoms.

** Data collected from non-treated patients of the Standard arm were considered at baseline and excluded for the rest of the follow-up.

RTX, rituximab.

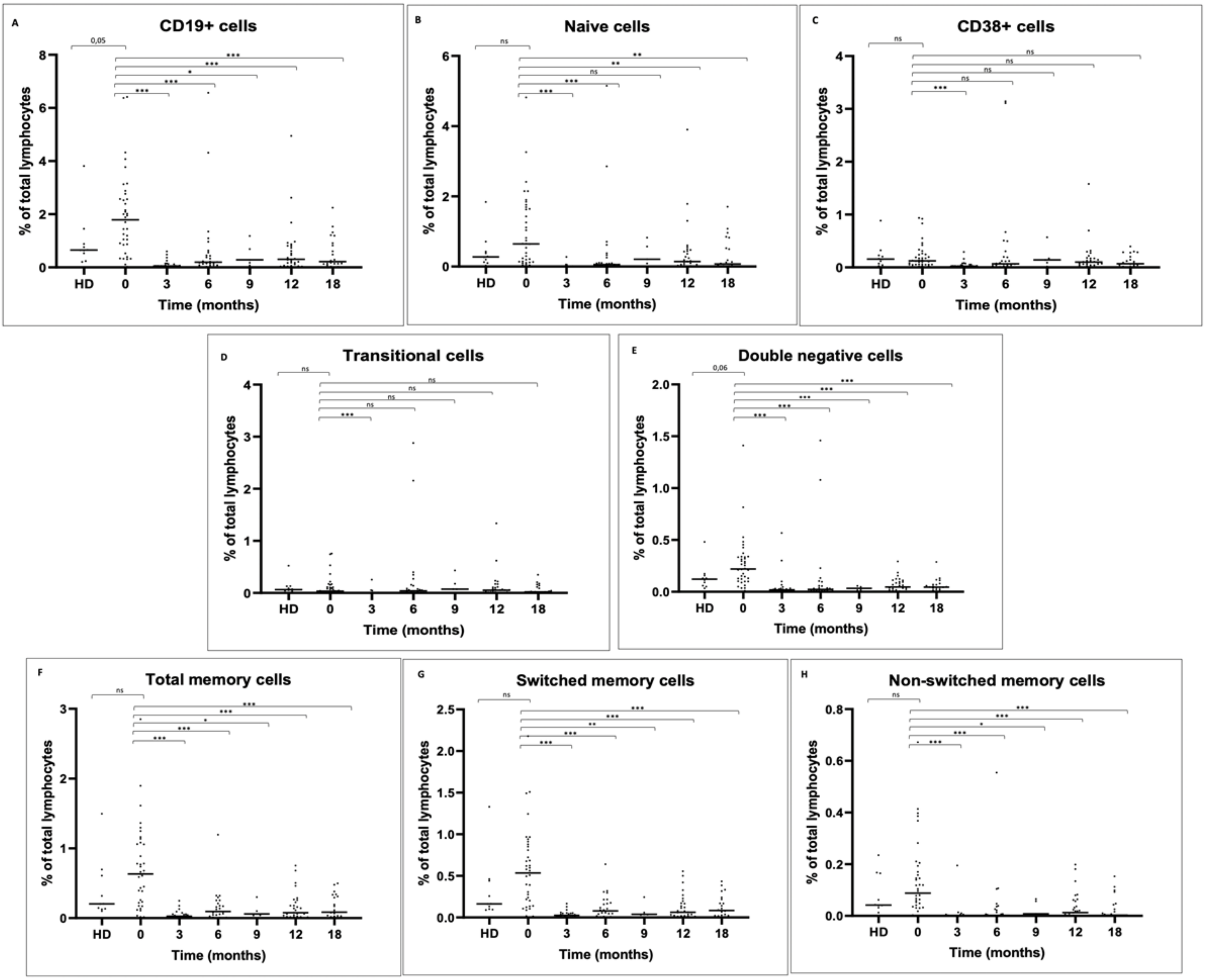

Figure S2: Multicolour flow cytometry analysis of B cell subpopulations at baseline and different time points after rituximab treatment in patients with MN.

(A–H). Baseline B-cell levels in patients (n = 39) were compared with baseline values from healthy donors (HD, n = 10) and with levels at various time points during follow-up in the 36 patients who received rituximab. (A) Gated CD19$^+$ B cells were identified based on surface marker expression, as depicted in the 'Materials and Methods' section. (B) naïve; (C) CD38$^+$; (D) transitional; (E) double negative; (F) total memory; (G) switched memory; (H) non-switched memory. At baseline, no difference was detected in the median percentage of naïve B cells (0.64% vs. 0.28%, p = 0.3), total memory cells (0.63% vs. 0.20%, p = 0.1), non-switched memory cells (0.09% vs. 0.04%, p = 0.4), CD38$^+$

cells (0.12% vs. 0.16%, p = 0.66), or transitional cells (median 0.03% vs. 0.06%, p = 0.4) between patients and HD. However, there was a tendency towards higher levels of total B cells (median 1.79% vs 0.65%, p = 0.05; A) and double-negative cells (0.22% vs 0.12%, p = 0.06; E). Baseline median percentage of B cells was never retrieved for any of the described subpopulations during follow-up, apart from $CD38^+$ cells at M6, M9, M12 and M18 and transitional cells at M6, M9, M12 and M18 (C and D). Double negative, memory, non-switched and switched memory cells recovered more slowly compared to B cell subpopulations previously described. Their percentages at M18 were still significantly lower than those at baseline (median 0.04% vs; 0.22%, 0.08% vs 0.63%, 0.003%, vs 0.09%, 0.08% vs 0.54%, respectively, all p values were <0.001) (E-H).

B cells were expressed as a percentage of total lymphocytes. Horizontal lines indicate the medians. Data were compared using the non-parametric, unpaired Mann–Whitney U test.

*p<0.05; **p<0.01; ***p<0.001; ns: not significant.

CD19+ cells at M6

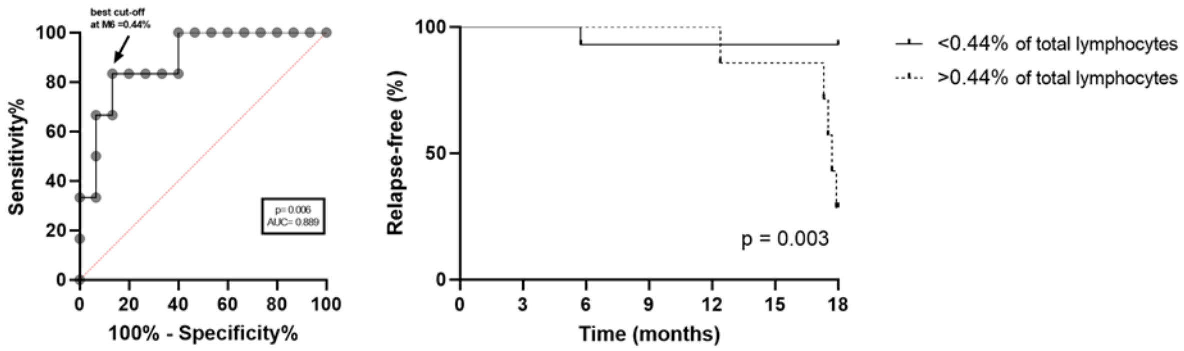

Naive cells at M6

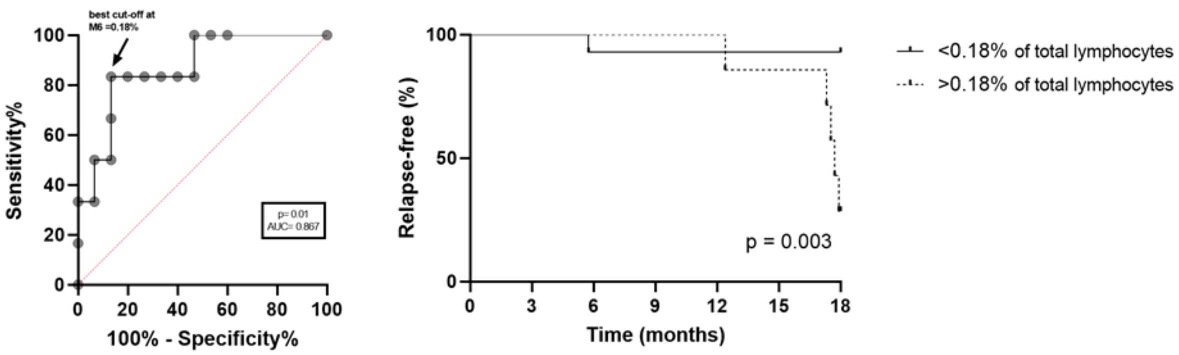

Double negative cells at M6

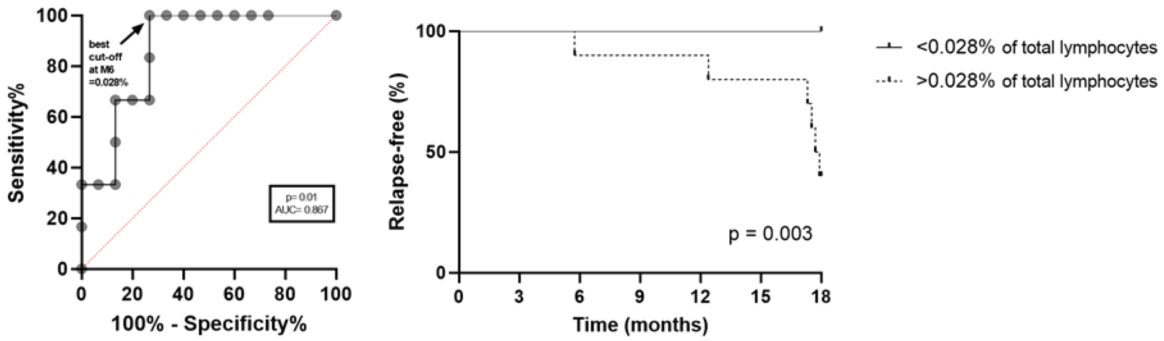

CD38+ cells at M6

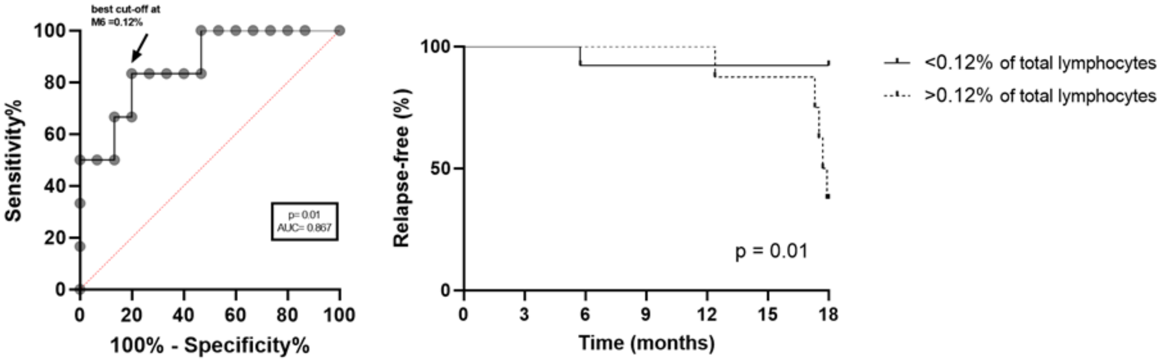

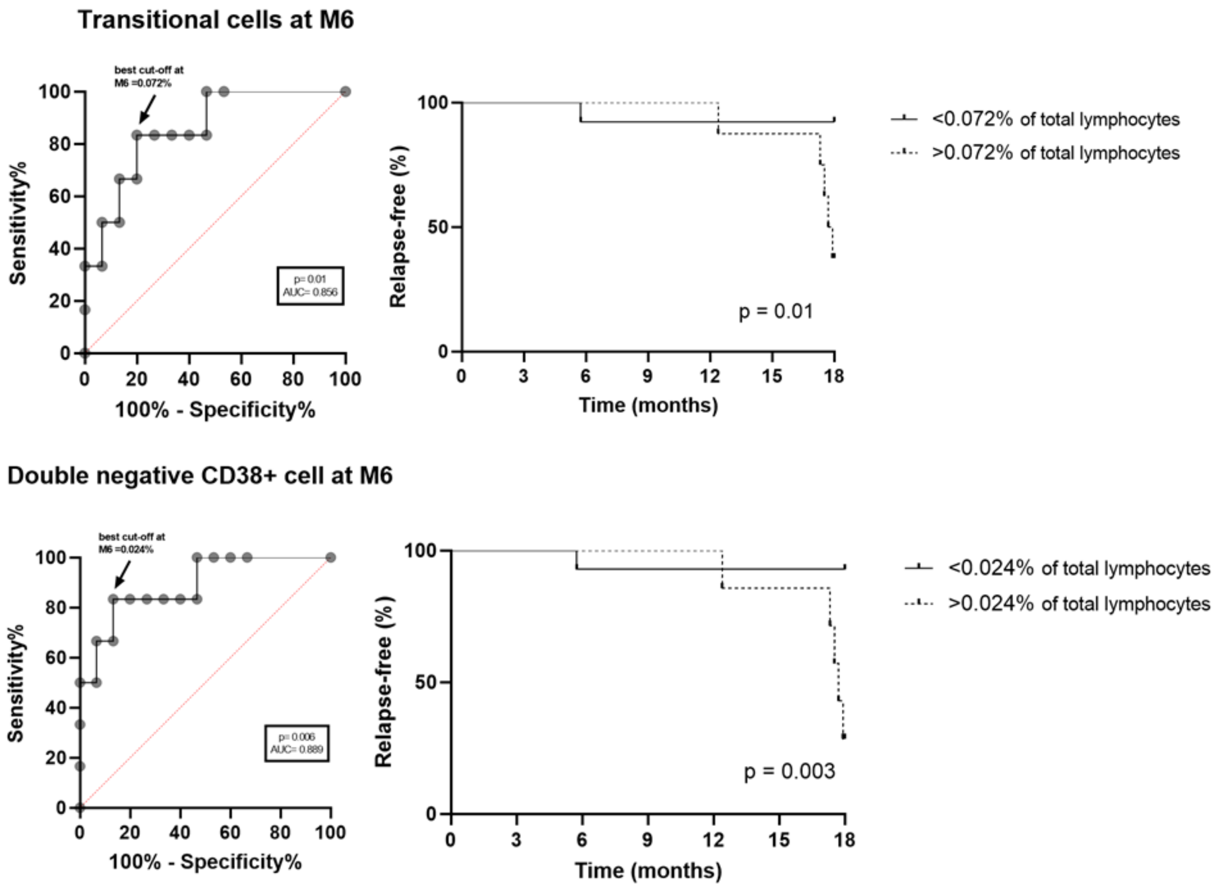

Figure S3: Determination of the optimal cut-offs for predicting relapse.

Reconstitution of $CD19^+$, naïve, double negative, $CD38^+$ transitional and double negative $CD38^+$ cells after rituximab treatment was predictive of relapse risk. The ROC curve analyzed these different B cell subsets and the values were expressed as a percentage of the total lymphocytes at six months after rituximab infusion and the risk of relapse within 18 months of treatment. The arrow indicates the optimal cut-off point for these subpopulations at M6 for predicting relapse. Survival analysis was used to compare patients with delayed (solid lines) and early (dashed lines) recovery of the described B cell subsets (by log-rank test).

AUC: area under the curve.

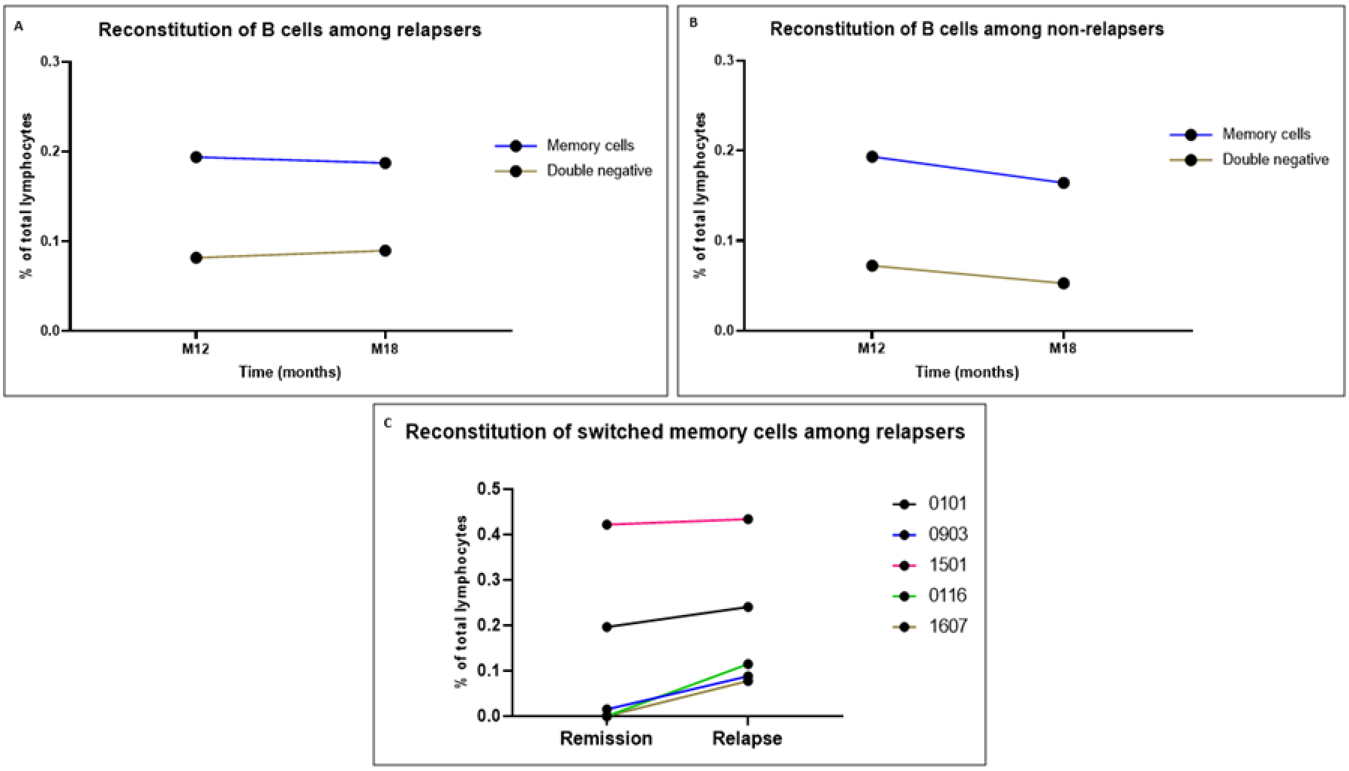

Figure S4: Reconstitution of memory and double-negative cells among relapsing and non-relapsing patients.

(A) The proportion of memory and double-negative cells among lymphocytes remained stable in relapsing patients 12 months after rituximab administration. (B) However, from this time point, the percentage of these subpopulations decreased in the non-relapsing group. (C) Among relapsing patients, the percentage of switched memory cells was higher at relapse than during remission. Each patient is represented by an inclusion number. Data are presented as means.
